# Evaluation of disability in patients exposed to fluoroquinolones

**DOI:** 10.1186/s40360-020-00415-4

**Published:** 2020-06-03

**Authors:** Marsha A. Wilcox, Angelina Villasis-Keever, Anthony G. Sena, Christopher Knoll, Daniel Fife

**Affiliations:** Janssen Research & Development, LLC, 1125 Trenton Harbourton Road, Titusville, NJ 08560 USA

**Keywords:** Luoroquinolone, Disability, Azithromycin, Sulfamethoxazole, Administrative data, Adverse events

## Abstract

**Background:**

Fluoroquinolones are used for conditions including sinusitis, bronchitis, and urinary tract infections. It has been suggested that exposure to fluoroquinolones for these conditions is associated with disability resulting from adverse events in 2 or more organ systems. The objectives were to: describe: 1) fluoroquinolone, azithromycin, and sulfamethoxazole / trimethoprim utilization for these infections; 2) the rate of disability associated with exposure to each of these antibiotic classes and adverse events in 2 or more system organ classes, and 3) compare outcome rates for each of the antibiotic classes.

**Methods:**

This study was conducted using administrative data to mitigate the limitations of spontaneous reports. The sampling frame was a U.S. population with both medical and disability insurance, including patients with the above uncomplicated infections who were prescribed the antibiotics of interest.

The primary outcome was an incident short-term disability claim associated with adverse events in 2 different organ systems within 120 days of exposure. A matched analysis was used to compare the outcome for patients receiving each of the drug classes.

**Results:**

After propensity score matching, there were 119,653 individuals in each of the exposure groups. There were 264 fluoroquinolone associated disability events and 243 azithromycin/ sulfamethoxazole associated disability events (relative risk =1.09 (95% CI: 0.92–1.30; calibrated *p* = 0.84)). The results were not significantly different from the null hypothesis of no difference between groups.

**Conclusion:**

Comparative assessments are difficult to conduct in spontaneous reports. This examination of disability associated with adverse events in different system organ classes showed no difference between fluoroquinolones and azithromycin or sulfamethoxazole in administrative data.

## Key points

1. It is possible to link disability and administrative claims datasets to evaluate disability as an outcome in a population with both medical and disability insurance.
2. This examination of disability associated with adverse events in more than one system organ class showed no difference between fluoroquinolones and azithromycin or sulfamethoxazole in administrative data.

## Background

Fluoroquinolones are a broad-spectrum class of antibiotics with high tissue distribution. They are indicated for a wide variety of infections and are among the most frequently prescribed antibiotics. An FDA safety review suggested that the use of fluoroquinolones is associated with disabling and potentially permanent adverse events (AEs) involving 2 or more organ systems that can occur together in the same patient. The FDA determined that the fluoroquinolones should be reserved for use in patients that have no other options for the following indications: acute sinusitis (AS), acute bacterial exacerbation of chronic obstructive pulmonary disease (AB) and uncomplicated urinary tract infection (UTI). In these indications, the FDA concluded that the risks of these serious side effects generally outweigh the benefits of the use of these antibiotics and all fluoroquinolone labels for systemic use were changed to reflect this recommendation [1].

Once the decision to prescribe an antimicrobial is made, the choice of antimicrobial should be based on an evaluation of both the benefits and adverse events of the antimicrobials available for the specific indication [2–11].. As with all antimicrobials, the use of fluoroquinolones is limited due to resistance and adverse events [12]. Practice guidelines and reviews by experts consider FQ as alternative to recommended therapy for the treatment of AS [2–4], AB [5–9] and UTI [10, 11].

### FDA adverse event reporting system

The possible association of the use of fluoroquinolones with disabling and potentially permanent adverse events (AEs) was identified from a review of the FDA Adverse Event Report System (FAERS) [1]. The FAERS is a database setup to support the FDA’s post-marketing surveillance program by recording adverse events spontaneously reported by consumers and health care professionals to the FDA or manufacturers [13]. This analysis of the spontaneous adverse event reports was conducted without an explicit prior hypothesis and without a comparator. As there is no measure of the total number of patients exposed to a particular drug in a spontaneously reported adverse event database, it is not possible to estimate the rate of adverse events. Janssen, a pharmaceutical company that has marketed a fluoroquinolone, is committed to examining the potential association in a study that would address some of these limitations in administrative claims data.

### Objectives

**Fig. 1:**
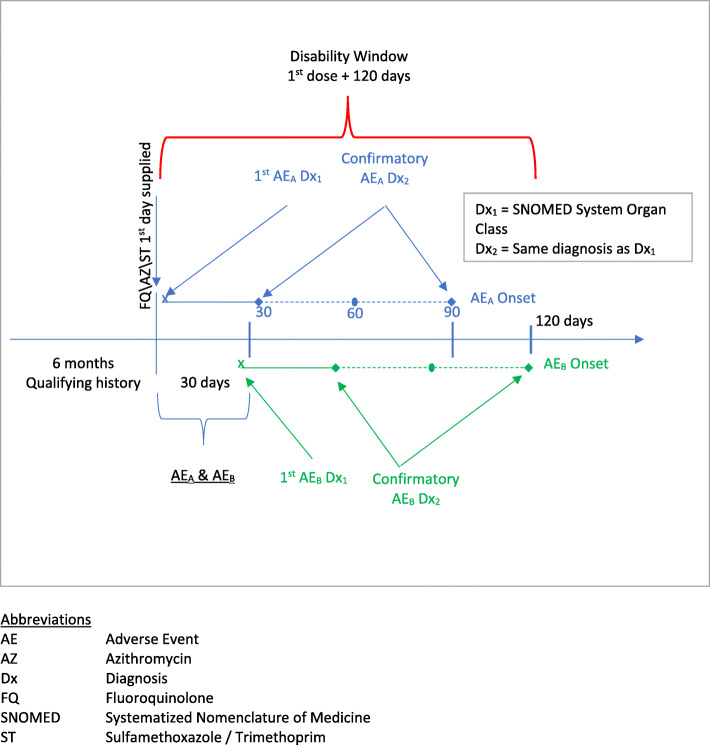
Study Design

The primary objectives of this study were to:

- Describe drug utilization for fluoroquinolone (FQ), azithromycin (AZ) for sinusitis and bronchitis, and sulfamethoxazole / trimethoprim (ST) for urinary tract infection in an entire health claims database and among those individuals in that database who eligible for short term disability benefits.
- Describe the rate of disability associated with 2 or more system organ class adverse events (SOC AEs) among individuals recently exposed FQs or AZ/ST for the indications described above, and
- Compare the rates of disability for AEs in 2 or more SOCs after recent exposure to FQs or AZ/ST for these indications (Fig. 1).

## Methods

### Sample

The sampling frame (the population from which the study patients arose) for this retrospective cohort study was men and women aged 18 through 65 years in a large, well characterized US commercially insured database, IBM MarketScan® Commercial database [CCAE] who were eligible for disability insurance and could be linked to the IBM MarketScan® Health and Productivity Management database (HPM) during all years for which such data were available, 2007 through 2015. Individuals entered the study on their first exposure to either an FQ or AZ/ST if at the time of that exposure they had been in the database for at least the past 6 months and remained in the database and were insured for disability for at least 120 days afterward, i.e. for the entire time at risk window (see Fig. 1). The date of that exposure was the individual’s index date.

#### *IBM MarketScan® Commercial Database* (CCAE)

IBM MarketScan® Commercial Database (CCAE) contains data from individuals enrolled in United States employer-sponsored insurance health plans. The database includes adjudicated health insurance claims (e.g. inpatient, outpatient, and outpatient pharmacy) as well as enrollment data from large employers and health plans who provide private healthcare coverage to employees, their spouses, and dependents. Additionally, it captures laboratory tests for a subset of the covered lives. This administrative claims database includes a variety of fee-for-service, preferred provider organizations, and capitated health plans.

The major data elements contained within this database are outpatient pharmacy dispensing claims (coded with National Drug Codes (NDC), inpatient and outpatient medical claims which provide procedure codes (coded in Current Procedural Terminology [CPT-4], Healthcare Common Procedure Coding System [HCPCs], International Classification of Diseases, Ninth Revision, Clinical Modification [ICD-9-CM] or ICD-10- Procedure Coding System [PCS]) and diagnosis codes (coded in ICD-9-CM or ICD-10-CM). The data also contain selected laboratory test results (those sent to a contracted thirds-party laboratory service provider) for a non-random sample of the population (coded with Logical Observation Identifiers Names and Codes [LOINC] codes).

#### *IBM MarketScan® Health and Productivity Management Database* (HPM)

IBM MarketScan® Health and Productivity Management Database (HPM) is a subset of the CCAE database, including employees for whom their employer provided information on absences, short-term disability (STD), and workers’ compensation. The data in HPM are linkable to the other IBM commercial datasets for these employees.

### Indications and exposures

The sample was limited to individuals who were diagnosed in an outpatient setting with uncomplicated acute bacterial sinusitis, or uncomplicated acute bronchitis and were dispensed an oral fluoroquinolone or azithromycin, but not both; and individuals who had an uncomplicated urinary tract infection (UTI) and were treated with an oral fluoroquinolone or sulfamethoxazole / trimethoprim (e.g. Bactrim), but not both. Exposure was required to occur within 30 days after the indication diagnosis. In case of disagreement between the diagnosis found in the CCAE database and that found in the HPM database, at the recommendation of the data owner, the former was used to identify the indication.

As was done by the FDA, we categorized AEs using the System Organ Class (SOC) in the MedDRA (Medical Dictionary for Regulatory Activities) medical terminology. The SOC is the highest level of term i.e. the level with the broadest terms in the classification. Examples of SOC’s include blood and lymphatic system disorders, cardiac disorders, ear and labyrinth disorders, endocrine disorders, eye disorders, musculoskeletal and connective tissue disorders, and psychiatric disorders [14]. The coding algorithms were designed by the authors to approximate the definitions used by the FDA in the analyses of the FAERS data. The algorithms were written into the protocol and the protocol was registered with ClinicalTrials.gov prior to conducting the study. Acute bronchitis was identified using code 466.0 (acute bronchitis). Acute sinusitis was identified using codes 461.0, 461.1, 461.2, 461.3, 461.8, and 461.9, (acute maxillary, frontal, ethmoidal, sphenoidal, other acute sinusitis, acute sinusitis not otherwise specified). Code 599.0 (urinary tract infection, site not specified) was used to identify urinary tract infections.

The first occurrence of the indication-exposure combination was used for this study. Each study participant qualified only for the first cohort for which he or she was eligible. The samples were mutually exclusive. That is, none of the FQ cohort was exposed to AZ/ST in the prior 6 months. Similarly, none of the AZ/ST cohort was exposed to FQ in the prior 6 months.

### General exclusions

Patients were excluded if they had any of the following conditions, procedures or exposures in the 6 months preceding the first qualifying dose of FQ or AZ/ST: fibromyalgia, rheumatoid arthritis, lupus, diabetes with complications, Lyme disease, multiple sclerosis, renal or hepatic impairment, HIV, joint replacement, or organ transplant; exposure to long-term oral steroid use (30 days or longer) or any cancer chemotherapy, any disability claim.

Condition-specific exclusions were imposed for events within the 3 months preceding the qualifying FQ or AZ/ST exposure. Patients with acute bronchitis were excluded if they had any of the following: hospitalization for: bronchitis, pneumonia, hypoxemia, respiratory insufficiency; outpatient diagnosis of pneumonia, hypoxemia or respiratory insufficiency. Exclusions for patients with acute sinusitis included: hospitalization for sinusitis or sinus surgery, outpatient sinus surgery or invasive outpatient procedure. Patients with UTI were excluded if they were hospitalized for a UTI, received a catheter or were diagnosed with urinary tract obstruction, pyelonephritis, renal abscess, malformation of the urinary tract, or chronic renal failure.

FQAD was described by FDA as a condition that arises in previously healthy patients after an uncomplicated infection. The exclusions were not for any hospitalization in the past 3 months, but for hospitalizations that were likely to be related to the infections and thus would make it likely that the patient’s infection did not qualify as an uncomplicated infection in a previously healthy person. These were not broad exclusions, but were exclusions included to ensure the patients in the study were candidates for the outcome of interest.

### Outcomes

The primary outcome was a disability claim in temporal proximity to confirmed AE’s in 2 different MedDRA SOCs among the 6 SOCs of interest (peripheral nervous system, neuropsychiatric, musculoskeletal, sensory, cardiovascular, skin). Disability was defined as an incident short-term disability claim in the HPM database observed within 120 days after the index date (Fig. 1). The disability claim was excluded if it was the continuation of a claim initiated prior to the index date.

Adverse events (AEs) of interest were reported in 2 or more system organ classes. The six categories used in the FDA report were mapped to 7 Medical Dictionary for Regulatory Activities (MedDRA) terms as follows (FDA-MedDRA): cardiovascular- cardiac disorders; sensory-ear and labyrinth disorders; sensory - eye disorders; musculoskeletal- musculoskeletal and connective tissue disorders; peripheral nervous -nervous system disorders; neuropsychiatric - psychiatric disorders; skin-skin and subcutaneous tissue disorders.

The IBM CCAE data were mapped to the Observational Medical Outcomes Partnership (OMOP) Common Data Model [15]. As part of this process, the OMOP vocabularies provide a standardized mapping between the ICD-9 codes provided in the IBM CCAE data and their respective related SNOMED standard codes. Additionally, the OMOP vocabulary provides a mapping between SNOMED and the MedDRA System Organ Classes used here. The design for mapping the IBM CCAE data set is maintained at https://github.com/OHDSI/ETL-CDMBuilder/tree/master/man/TRUVEN_CCAE_MDCR_MDCD.

### Negative control outcomes

We chose 45 negative control conditions, conditions believed not to be causally associated with either of the exposure cohorts based on a review of published literature, product labeling and spontaneous adverse event reporting (Supplemental material) to identify residual systematic error in the database or study design, and to empirically calibrate *p*-values for systematic error. For each negative control outcome, we assumed a priori that the true odds ratio (OR) for the outcome was the null value of 1. We then applied the same analysis used for the study outcomes to each negative control outcome. The difference between the estimated OR for the negative control condition and the expected null value represented an estimate of the systematic error present for that outcome. The distribution of the error estimates from the negative controls was used as the empirical null distribution. We used this distribution to compute a calibrated *p*-value for each outcome [16, 17].

### Time-at-risk periods

Our choice of time-at-risk periods was informed by the Briefing Book from the FDA Advisory Panel in 2015 (page 24) [1]:

“The mean and median time to onset of adverse events was 5.4 days and 3 days, respectively. However, the range was very wide, from 1 hour after taking the first dose to 90 days after the drug was discontinued. In almost half of the cases (48%), the onset was rapid, occurring after one or two doses of the drug. In 12% of the cases, the onset occurred more than 10 days after starting the fluoroquinolone, which in most cases would have been after fluoroquinolone therapy had ended.”

If the symptoms are disabling, they should lead to 2 medical encounters within 90 days, and in some cases, much sooner. The appropriate sensitivity analysis, therefore, focused on a shorter, 90-day, at-risk-period.

Qualifying confirmed AEs were incident within 30 days of the first day supplied of the exposure drug with a duration of 30 days or longer. In our primary analysis, in order to be “confirmed” the same diagnosis was required to be observed 30–90 days after the incident diagnosis (Fig. 1). To assess the effect of some of our model assumptions about the length of time from exposure to AE we conducted a sensitivity analysis in which we shortened the window for the confirmatory diagnosis from 60 days to 30 days.

### Comparators

The purpose of this study was to examine FQAD among patients being treated for uncomplicated acute sinusitis, acute bronchitis, or acute urinary tract infection in an insured population in the U.S. If the occurrence of 2 SOC AEs and disability is associated with FQs, an analogous condition should not also be satisfied for people who received other types of antibiotics, e.g., azithromycin (AZ). Since AZ is not typically used to treat UTI, used Sulfamethoxazole / Trimethoprim (e.g. Bactrim) (ST) as the comparator for that condition.

### Statistical analyses

Crude incidence rates of both outcomes (primary and sensitivity) were estimated within each cohort as the number of individuals with the outcome during each time-at-risk window, divided by the total time-at-risk.

Propensity score adjustment was used as an analytic strategy to reduce potential confounding as the result of imbalance in baseline covariates between the target (FQ) and comparator (AZ/ST) cohorts. The propensity score was the probability of a patient being classified in the target cohort vs. the comparator cohort, given a set of observed covariates. The propensity score was estimated for each patient using the predicted probability from a regularized logistic regression model fit with a Laplace prior (LASSO) and the regularization hyperparameter selected by optimizing the likelihood in a 10-fold cross validation, using a starting variance of 0.01 and a tolerance of 2e-7 [17]. The classes of baseline covariates included in the propensity score model included demographics, diagnoses, drug exposures, and procedures observed in the 30-day, 6-month, and 1-year windows prior to antibiotic exposure [16]. A list of the covariates used in the propensity score can be found in the Supplemental Material.

Propensity score estimates were used to restrict the cohorts through patient trimming. Patients were excluded if their predicted probability was less than 5% or greater than 95% of the propensity score distribution across both cohorts. Patients in the target cohort were matched to patients in the comparator cohort using 1:1 matching with a greedy matching algorithm and a caliper of 0.25 of the standard deviation of the propensity score distribution. Standardized mean difference was used as a metric to evaluate the performance of propensity score adjustment.

### Comparison

The outcome model, a conditional Logistic regression, was summarized with the odds ratio and associated 95% confidence interval. We report effect estimates with nominal *p*-values and empirically calibrated *p*-values [18]. Since the empirical calibration captured systematic error observed from 45 negative controls (Supplemental Material), this statistic was our a priori primary decision criterion for determining statistical significance, including in scenarios where the nominal *p*-value and calibrated *p*-values might have been inconsistent.

### Statistical power

Given matched sample sizes of 119,653, α = 0.05, prevalence of 0.002, we had 80% power to detect an odds ratio of 1.25 or greater.

## Results

### Sample before matching

**Fig. 2:**
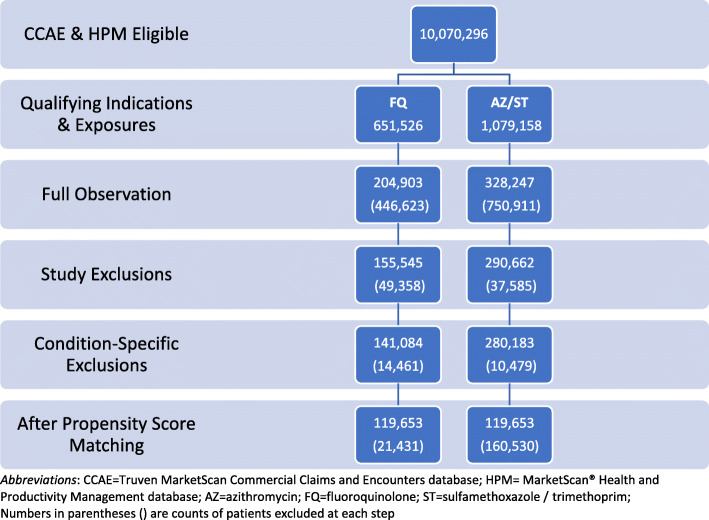
Sample Disposition

There were more than 10 million (10,070,296) distinct individuals in the CCAE database who were also eligible for disability insurance. Among those, 651,526 individuals were exposed to FQ for any of the qualifying indications; 1,079,158 were exposed to AZ/ST. The number with full observation time was 204,903 for FQ and 328,247 for AZ/ST. After study and condition-specific exclusions, there were 141,084 individuals in the FQ and 280,183 in the AZ/ST unmatched cohorts (Fig. 2).

### Sample after matching

**Fig. 3:**
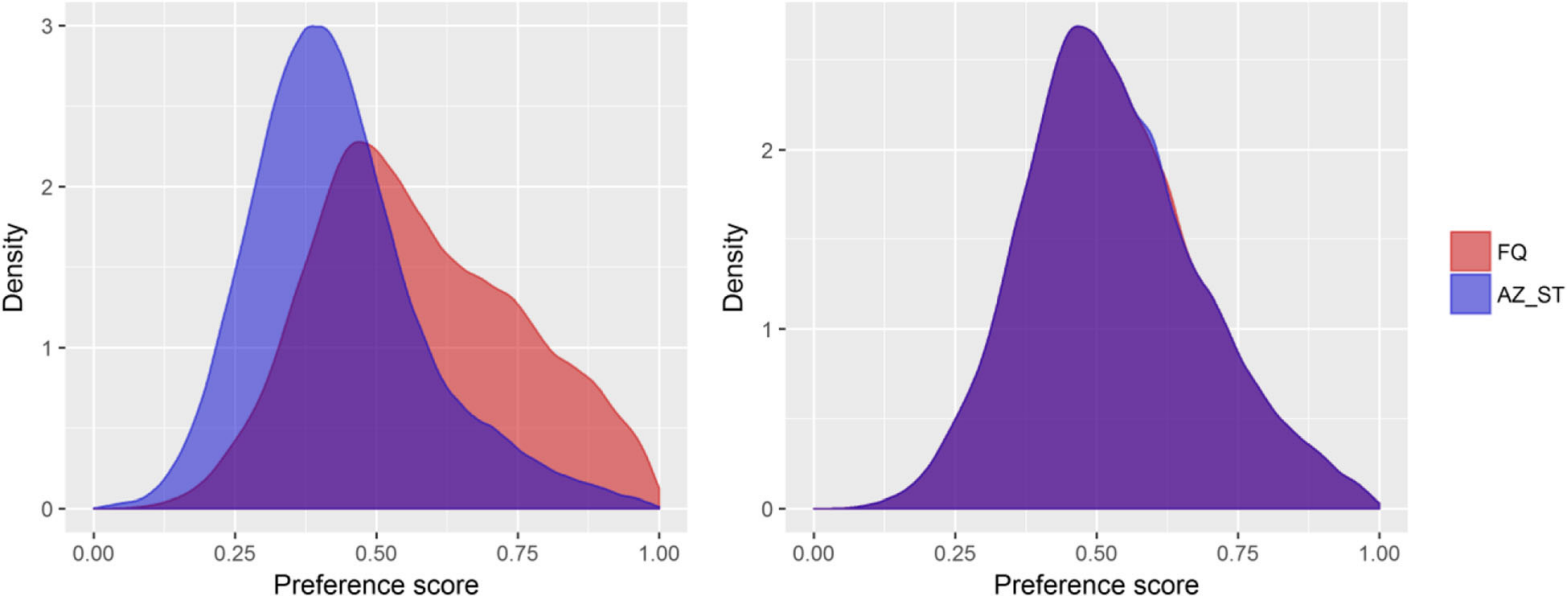
Preference Score Distribution Before and After Matching

After propensity score matching, there were 119,653 individuals in each of the exposure groups. Figure 2 shows the sample disposition at each step in the sample selection process. Details about the propensity score model can be found in the Supplemental Material. The standardized difference between groups ranged from −.06 (30–34 age group) to 0.23 (dysuria) before matching. After matching, all standardized differences were below 0.1. The range was − 0.03 (female gender) to 0.02 (dysuria) (Table 1). The preference score is a transformation of the propensity score that adjusts for differences in the sizes of the two treatment groups. The preference score plot shows the distribution of the score in each of the samples before and after matching (Fig. 3). Overlap of the distributions indicates subjects in the two groups were similar in terms of their predicted probability of receiving one treatment over the other.

**Table 1:** Sample characteristics before and after matching

Characteristic	Before matching			After matching		
N	FQ	AZ/ST		FQ	AZ/ST	
	155,776	294,663		119,653	119,653	
	Percent		Standardized Difference	Percent		Standardized Difference
**Age group**						
**15–19**	0	0	*0*	0	0	*0*
**20–24**	0.9	1	*−0.01*	0.9	0.9	*0*
**25–29**	5.4	6.3	*−0.04*	5.7	5.5	*0.01*
**30–34**	9	10.8	*−0.06*	9.4	9.1	*0.01*
**35–39**	12.3	14	*−0.05*	12.6	12.3	*0.01*
**40–44**	15.4	16.1	*−0.02*	15.5	15.2	*0.01*
**45–49**	17.5	17	*0.01*	17.4	17.5	*0*
**50–54**	18.3	17	*0.04*	18.1	18.4	*−0.01*
**55–59**	14.9	12.8	*0.06*	14.4	14.9	*−0.01*
**60–64**	6.2	4.9	*0.06*	5.9	6.2	*−0.01*
**65–69**	0.1	0	*0.01*	0.1	0.1	*0*
**Female gender**	59.1	49.3	*0.2*	57	58.6	*−0.03*
**Medical history**						
**Dysuria**	6	1.7	*0.23*	3.9	3.5	*0.02*
**Hematuria syndrome**	3.2	0.9	*0.16*	1.9	1.7	*0.01*
**Medication use**						
**Phenazopyridine hydrochloride 200 mg oral tablet**	5.3	1.3	*0.23*	3.1	2.8	*0.02*
**Nitrofurantoin, Macrocrystals 25 mg/ Nitrofurantoin, Monohydrate 75 mg oral capsule**	9.9	5.6	*0.16*	7.9	7.9	*0*
**Amoxicillin 875 mg / Clavulanate 125 mg oral tablet**	9.2	4.9	*0.17*	7.6	8.1	*−0.02*

### Antibiotic use for indications of interest after matching

Azithromycin was used more than any of the fluoroquinolones to treat sinusitis (59,501 vs. 48,170). Among the fluoroquinolones, levofloxacin was most often used for this indication. Similarly, azithromycin was used more often to treat bronchitis (47,933 vs. 31,503), with levofloxacin the most often used fluoroquinolone. For UTI, fluoroquinolones were prescribed more often than sulfamethoxazole / trimethoprim, (57,676 vs. 22,700). Ciprofloxacin was used ten times more often than the next most often used FQ, levofloxacin (Table 2). Ciprofloxacin and levofloxacin accounted for the majority of fluoroquinolone use overall. Levofloxacin was used most for the treatment of sinusitis and bronchitis, while ciprofloxacin was used most for urinary tract infections.

**Table 2:** Indications of interest treated by antibiotics of interest after matching

	Sinusitis	Bronchitis	UTI^a^	Any the Conditions
Levofloxacin	31,065	21,723	5170	49,923
Ciprofloxacin	8535	4226	52,307	56,792
Moxifloxacin	8178	5143	175	12,117
Ofloxacin	7	4	45	52
Gemifloxacin	420	424	13	785
Gatifloxacin	1	0	1	2
Norfloxacin	0	1	4	5
FQ Total	48,170	31,503	57,676	119,653
AZ/ST	59,501	47,933	22,700	119,653
*AZ*^*b*^	*59,206*	*47,886*	*461*	*99,871*
*ST*^*b*^	*328*	*75*	*22,255*	*19,782*

Abbreviations: *AZ* Azithromycin, *FQ* Fluoroquinolone, *ST* Sulfamethoxazole / Trimethoprim, *UTI* Urinary tract infection

^a^UTI indication was treated by ST

^b^AZ exposure required indication of sinusitis/bronchitis and ST required UTI. Other indications may co-occur with these treatments

### Antibiotic-associated disability (FQ AD/AZST AD)

There were 264 cases of FQAD. Among those, 117 were exposed to levofloxacin, 111 to ciprofloxacin, 34 to moxifloxacin, and 2 to Gemifloxacin (Table 3). There were 243 cases of antibiotic associated disability among those exposed to azithromycin or sulfamethoxazole / trimethoprim.

**Table 3:** AE count and disability for each fluoroquinolone (counts)^a^

		1 confirmed qualifying AE	1 confirmed qualifying AE + Disability	2+ confirmed qualifying AEs	2 + confirmed qualifying AEs + Disability
	Count Used for Any Condition	Count (Row %)			
Levofloxacin	49,923	7559 (15.1%)	472 (0.9%)	1328 (2.7%)	117 (0.2%)
Ciprofloxacin	56,792	7062 (12.4)	530 (0.9)	1109 (2.0)	111 (0.2)
Moxifloxacin	12,117	1863 (15.4)	105 (0.9)	281 (2.3)	34 (0.3)
Ofloxacin	52	6 (11.5)	0 (0.0)	1 (1.9)	0 (0.0)
Gemifloxacin	785	110 (14.0)	5 (0.6)	16 (2.0)	2 (0.3)
Gatifloxacin	2	0	0 (0.0)	0 (0.0)	0 (0.0)
Norfloxacin	5	0	0 (0.0)	0 (0.0)	0 (0.0)
TOTAL	119,676	16,600	1112	2735	264

Abbreviations: *AE* Adverse event

^a^Counts above are not mutually exclusive

Descriptive statistics about the cases in both cohorts can be found in Table 4. The median age of cases in the FQ cohort was 49; 51 in the AZ/ST cohort. Women comprised 55.3% of the FQ cohort; 62.6% of the AZ/ST group. In the non-elderly population, UTI’s are more common in women than in men, and ST is very often used to treat uncomplicated UTI’s.

**Table 4:** Descriptive statistics for antibiotic associated disability cases

		FQ AD		AZ/ST AD	
		*N* = 264		*N* = 243	
		Count	%	Count	%
**Age**	*Mean (sd)*	*48.5 (8.2)*		*48.8 (8.7)*	
	*Median*	*49*		*51*	
	*Range*	*26–64*		*24–64*	
	18–29 years	5	1.9	6	2.5
	30–59 years	238	90.2	218	89.7
	> = 60 years	21	8.0	19	7.8
**Gender**	Female	146	55.3	152	62.6
	Male	118	44.7	91	37.4
Non-UTI Cases^a^	Total	164	62.1	207	85.2
	Female	73	44.5	122	58.9
	Male	91	55.5	85	41.1
**Indication**	Sinusitis	97	33.1	96	35.3
	Bronchitis	50	17.1	100	36.8
	Cystitis/UTI	100	34.1	36	13.2
	Sinusitis/bronchitis	15	5.1	9	3.3
	Bronchitis/UTI	2	0.7	2	0.7
**Days to AE Onset**	*Mean (sd)*	*9.7 (7.1)*		*10.5 (7.8)*	
	*Median*	*8*		*8*	
	*Range*	*1–30*		*1–29*	
	1–2 days	41	15.5	38	15.6
	3–4 days	33	12.5	29	11.9
	5–10 days	88	33.3	77	31.7
	> 10 days	102	38.6	99	40.7
**Days to Confirmatory Diagnosis**	*Mean (sd)*	*46.7 (16.0)*		*49.0 (16.0)*	
Range 30–90 days	*Median*	*42*		*44*	

Abbreviations: *AZ* Azithromycin, *FQ* Fluoroquinolone, *ST* Sulfamethoxazole / Trimethoprim, *UTI* Urinary tract infection

^a^The count of Non-UTI cases includes indications for bronchitis and sinusitis. When these indications occurred in combination with UTI, they were included in this count

Roughly 1/3 of each group were treated for sinusitis. In the FQ cohort, 17% were treated for bronchitis; the number was nearly twice that (37%) for the AZ/ST group. Close to 1/3 of the FQ group were treated for cystitis or a UTI; only 13% in the AZ/ST group were treated for this indication. Among those exposed to FQ, 55.3% were women, the same was true for 62.6% of the AZ/ST cohort. The median time to AE onset was 8 days for both groups. The median time to confirmatory diagnosis was 42 days for the FQ group; 44 days in the AZ/ST group. The range for both groups was 30–90 days.

### Comparison of rates of FQ AD with AZ/ST AD

There were 264 FQAD events and 243 AZ/ST events in the matched samples (Table 5). The observed crude odds ratio was 1.09 (95% CI: 0.92–1.30). The *p*-value for the adjusted odds ratio was *p* = 0.35; the calibrated *p*-value was *p* = 0.84. Calibration results can be found in the Supplemental Material. The results were not significantly different from the null hypothesis of no difference between groups.

**Table 5:** Outcome - crude and adjusted odds ratios (ORs)

	FQ	AZ/ST	FQ	AZ/ST	Crude OR	Adjusted OR
	(N, col. %)	(N, col. %)	(95% CI)	(95% CI)	(95% CI)	(95% CI)
N	119,653	119,653				
> = 2 SOC AEs + Disability	264	243	0.002	0.002	1.09	1.09
	0.22%	0.20%	(0.002–0.002)	(0.002–0.002)	(0.92–1.30)	(0.91–1.30)
						*p* = 0.35
						*calibrated p = 0.84*
**Sensitivity Analyses**						
> = 2 SOC AEs + Disability	205	182	0.002	0.002	1.13	1.13
	0.17%	0.15%	(0.001–0.002)	(0.001–0.002)	(0.92–1.37)	(0.92–1.38)
						*p* = 0.24
						*calibrated p = 0.89*

Abbreviations: *AZ* Azithromycin, *FQ* Fluoroquinolone, *ST* Sulfamethoxazole / Trimethoprim

Table 6 shows the distribution of SOC AEs in the cases. Cases in the FQ group had an average of 2.66 AEs. The average was 2.64 in the AZ/ST cohort.

**Table 6:** System Organ Class AEs in the Cases

	Cases (At least 2 SOCs)	
System Organ Class	FQ AD	AZ/ST AD
	***n*** = 264	***n*** = 243
Peripheral nervous	131 (0.11%)	87 (0.07%)
Neuropsychiatric	103 (0.09%)	87 (0.07%)
Musculoskeletal	75 (0.06%)	139 (0.12%)
Sensory	161 (0.13%)	197 (0.16%)
Cardiovascular	209 (0.17%)	115 (0.10%)
Skin	24 (0.02%)	16 (0.01%)
*Total*	*703*	*641*
*Average per case*	*2.66*	*2.64*

### Sensitivity analysis

In the sensitivity analysis, restricting the observation window to 30 days, there were 205 events in the FQ cohort and 182 events in the AZ/ST cohort with an adjusted odds ratio of 1.13. The *p*-value for the adjusted odds ratio was *p* = 0.24; the calibrated *p*-value was *p* = 0.89. The results for the sensitivity analysis were not different from the hypothesis of no difference between groups.

While the counts were lower, the inference was the same; no difference between groups in the incidence of antibiotic-associated disability (Table 5). Detailed results from the sensitivity analyses can be found in the Supplemental Material.

We examined the distribution of time to the second, confirmatory, diagnosis in both our primary 120-day window and the 90-day window in the sensitivity analysis. The median time to confirmatory diagnosis was 42/44 days (FQAD/AZSTAD) in the primary 120-day analysis and 37/40 days in the 90-day sensitivity analysis.

## Limitations

There were several limitations in this work. The source population was limited to administrative healthcare claims among a privately insured population with disability insurance. Our definition of disability required employment and therefore excluded the elderly and the unemployed populations. The disability data were not perfectly matched to the medical claims because not all the CCAE database contributors were able to supply all types of HPM data for every data year.

The average dwell time in such databases is approximately 2 years. Qualifying events that began prior to the insurance coverage or persisted afterward were censored. Similarly, events that began prior to the observation period and exposures that occurred prior to the observation period were missed.

We necessarily made assumptions about the allowable time between the first and second diagnosis and the allowable time for filing a claim. Though we did sensitivity analyses, it remains possible that different choices of these times would have yielded different estimates of the relative risk. We conducted a sensitivity analysis about the time to qualifying adverse events. The point estimate was similar and the inference was the same.

There are several ways in which our study design could introduce bias. First, we require patients to have 120 days of observation post exposure. Patients for whom the AEs were severe may have died or lost their insurance and would be lost to the study. In our effort to replicate the FDA study, we required 2 AEs prior to the disability claim. Patients with mortality related to a single AE would not have the opportunity to be counted in our work. Further, we required a confirmation of each of the AEs during a 30-day window, thereby introducing immortal time bias. This too, has the potential for biasing our findings.”

Our findings should be interpreted in light of the limitations inherent in claims-based analyses. The results of this work may not be generalizable to populations not included in the study (e.g., patients who are uninsured).

## Discussion

The benefit-risk profile of antibiotics is relatively easy to discern when the infection is severe and the burden of disease great. It can be a more challenging question when the infection is less severe and is uncomplicated. It was in this context the idea of fluoroquinolone-associated disability arose in spontaneous report data. While these data include a reporter, an outcome and a drug, the reports do not have a known denominator with which to estimate and compare rates. We sought to evaluate the characteristics of FQAD, including the question of whether it is unique to FQ’s or whether a similar pattern of adverse events and disability occurs with the use of other antibiotics used to treat the same conditions. We conducted the work in a large US administrative claims database in which the denominator would be known. To that end, we compared the disability rate in fluoroquinolones with the rates observed with the use of AZ/ST, when prescribed for the indications of interest.

Current FDA labeling for fluoroquinolones carries a Boxed Warning that appears to be based on FQAD in that it speaks of disabling and potentially irreversible serious adverse reactions that have occurred together, names several body systems that may be affected, and, for each fluoroquinolone, states that for treatment of uncomplicated urinary tract infection, acute bacterial exacerbation of chronic bronchitis, or acute bacterial sinusitis the use of fluoroquinolone should be reserved for patients who have no alternative options. FDA announcements such as the one at https://www.fda.gov/drugs/information-drug-class/fda-approves-safety-labeling-changes-fluoroquinolones, also suggest that this language is essentially warning about FQAD.

The present study offers evidence that such serious disabling and potentially irreversible adverse reactions that have occurred together are infrequent (incidence of 0.2%) and not unique to fluoroquinolones but also occur at approximately the same frequency after exposure to azithromycin sulfamethoxazole / trimethoprim for the same three indications among new users in the first 30 days after the start of exposure.

Our findings have important implications for understanding the safety profile of antimicrobials. When a decision to prescribe antimicrobials is made, the choice of antimicrobial should be based on an evaluation of both the potential benefits and adverse events of the antimicrobials available for the specific indication. For some indications, the benefit of the use of antimicrobials is limited: in acute sinusitis where the prevalence of bacterial infection is only 2–10%, and up to 80% of cases improve spontaneously; in mild cases of acute bacterial exacerbation of chronic bronchitis the effect of antibacterial drugs is modest, and its routine use is therefore not recommended. In these cases, the use of any antimicrobial should be limited to those cases where there is clear evidence of potential benefit.

## Conclusion

In propensity-score matched sample from a defined US working population with disability insurance, we found no difference between the incidence of disability associated with AE’s in two SOCs between those exposed to FQ’s and those exposed to AZ/ST.

## Supplementary information

**Additional file 1: **Fluoroquinolone and Disability – Negative Control Outcomes and Propensity Score Model. The file contains a list of the negative control outcomes used and the resultant *p*-value calibration. The file also contains a description of covariates evaluated for inclusion in the propensity score model and a reference to the accompanying excel file containing model parameters.

**Additional file 2:.** Model parameters for propensity scores. Model details for propensity scores.

## Data Availability

The data that support the findings of this study are available from IBM MarketScan® but restrictions apply to the availability of these data, which were used under license for the current study, and so are not publicly available. Data are however available from the authors upon reasonable request and with permission of IBM MarketScan®.

## Abbreviations

ABECB: Acute bacterial exacerbation of chronic obstructive pulmonary disease
AE: Adverse Event
AS: Acute sinusitis
AZ: Azithromycin
CCAE: IBM MarketScan® Commercial database
CPT: Current Procedural Terminology
Dx: Diagnosis
FAERS: FDA Adverse Event Reporting System
NFQ: Fluoroquinolone
HCPCS: Healthcare Common Procedure Coding System
HPM: Health and Productivity Management Database
ICD-9-CM: International Classification of Diseases, 9th Revision, Clinical Modification
ICD-10-PCS: International Classification of Disease -10th Revisions, Procedure Coding System
LOINC: Logical Observation Identifiers Names and Codes
OMOP: Observational Medical Outcomes Partnership
NDC: National Drug Codes
SNOMED: Systematized Nomenclature of Medicine
SOC: System organ class
ST: Sulfamethoxazole / Trimethoprim
UTI: Urinary tract infection
