# Supplementary material for: Evaluation of disability in patients exposed to fluoroquinolones
Source: BMC Pharmacol Toxicol. 2020 Jun 3;21:40. doi: 10.1186/s40360-020-00415-4 (PMC7268406; doi:10.1186/s40360-020-00415-4)
Supplement: Supplementary file 1 — Additional file 1: Fluoroquinolone and Disability – Negative Control Outcomes and Propensity Score Model. The file contains a list of the negative control outcomes used and the resultant p-value calibration. The file also contains a description of covariates evaluated for inclusion in the propensity score model and a reference to the accompanying excel file containing model parameters. [file 40360_2020_415_MOESM1_ESM.docx]

**Negative Outcome Analysis**

Negative Control Outcome List

| Abnormality of organs AND/OR soft tissues of pelvis affecting pregnancy | Hemospermia | Neonatal Abstinence Syndrome |
| --- | --- | --- |
| Acidemia | Hernia of abdominal cavity | Neoplasm of uncertain behavior of endocrine gland |
| Benign neoplasm of endocrine gland | Histoplasmosis | Nocturia |
| Complication associated with device | Human papilloma virus infection | Obesity |
| Complication of anesthesia | Hyperplasia of prostate | Occlusion of ureter |
| Deficiency of macronutrients | Hypovolemic shock | Open wound |
| Diabetic renal disease | Impaction of intestine | Poisoning |
| Disease due to Orthopoxvirus | Inflammatory disorder of breast | Post-traumatic wound infection |
| Effects of heat AND/OR light | Injury of abdomen | Puberty bleeding |
| Effects of hunger | Injury of chest wall | Rectal prolapse |
| Fetal condition affecting obstetrical care of mother | Injury of face | Simple goiter |
| Fibrocystic disease of breast | Intracranial injury | Toxic nephropathy |
| Finding of gravid uterus | Malignant immunoproliferative disease | Ulcer of anorectal structure |
| Gammopathy | Malignant neoplasm of endocrine gland | Upper gastrointestinal hemorrhage |
| Gastrointestinal and digestive injury | Malignant neoplasm of genital structure | Vesicoureteric reflux |
|  |  | Viremia |

## Negative control estimates


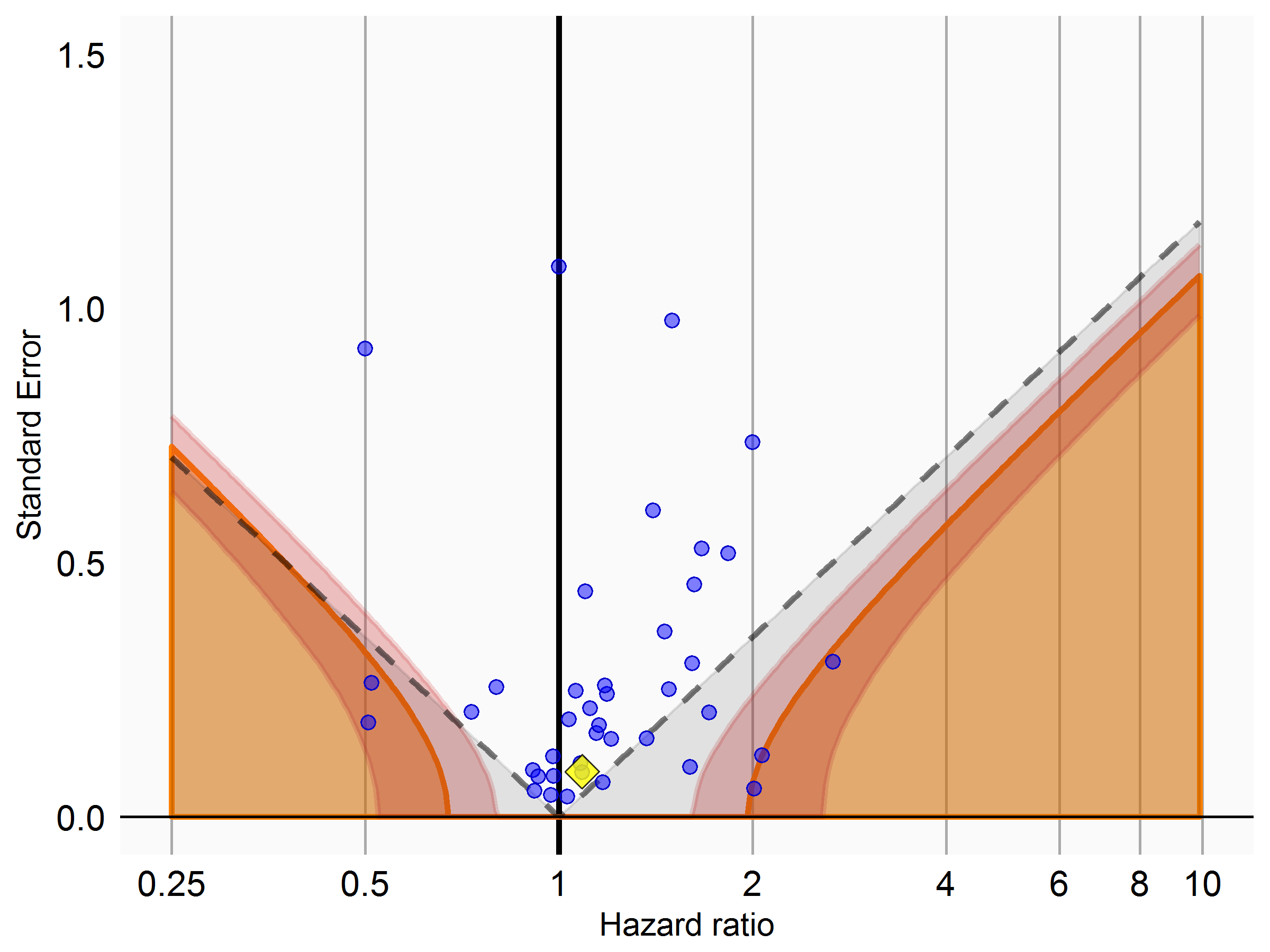


The plot above provides a graphical representation of the p-value calibration using the negative control outcomes. Each blue dot represents the estimated odds ratio (x-axis) and standard error (y-axis) for each of the negative control outcomes. Estimates below the dashed line have an uncalibrated (unadjusted) p < .05. Estimates in the orange area have calibrated p < .05 The red band indicates the 95% credible interval around the boundary of the orange area. Think of the credible interval as a confidence interval for the calibrated area. The orange area is the area of statistical significance by our criteria.

For example, the red circled data point on the left side of the graph is the estimate for one of the negative controls that falls into the orange area making it statistically significant after calibration. The green circled data point on the right side is the estimate for a negative control that is statistically significant before calibration but after calibration it loses statistical significance. The yellow diamond indicates the outcome of interest for our study which falls outside both the traditional and calibrated p-value areas indicating that it is not statistically significant.

# **Propensity Score**

## Covariates evaluated for inclusion in the propensity score model

- Gender
- Age (grouped in 5-year buckets)
- Year of Index
- All conditions in the preceding 30d, 180d and 365d
- All drug exposures in the patient history
- All procedures in the preceding 30 days and 180d

## Propensity Score Model

See accompanying Excel file: “Propensity Score Model – FQ Disability.xlxs”
